# Supplementary material for: A Cleanable Self-Assembled Nano-SiO2/(PTFE/PEI)n/PPS Composite Filter Medium for High-Efficiency Fine Particulate Filtration
Source: Materials (Basel). 2021 Dec 18;14(24):7853. doi: 10.3390/ma14247853 (PMC8706235; doi:10.3390/ma14247853)
Supplement: Supplementary file 1 [file materials-14-07853-s001.zip › materials-1457276-supplementary.pdf]

materials

MDPI

Supplementary Materials

A Cleanable Self-assembled Nano-SiO_2/(PTFE/PEI)$_n$/PPS Composite Filter Medium for High-efficiency Fine Particulate Filtration

Yan Luo[1,2,*], Zhongyun Shen[2,*], Zhihao Ma[2], Hongfeng Chen[1,2], Xiaodong Wang[2], Minger Luo[2], Ran Wang[3] and Jianguo Huang[1,*]

[1] Department of Chemistry, Zhejiang University, Hangzhou 310027, China; 0620158@zju.edu.cn (H.C.)
[2] Shaoxing Testing Institute of Quality and Technical Supervision, Market Supervision Administration of Shaoxing Municipahty, Shaoxing 312366, China; mzh@stiq.org (Z.M.); wangxd@stiq.org (X.W.); sxzjjd@stiq.org (M.L.)
[3] CAM-China Productivity Center for Machinery, China Academy of Machinery Science and Technology, Beijing 100044, China; wangran@pcmi.com.cn (R.W.)
* Correspondence: 0619739@zju.edu.cn (Y.L.); szy@stiq.org (Z.S.); jghuang@zju.edu.cn (J.H.); Tel.: +86-571-8795-1202 (J.H.)

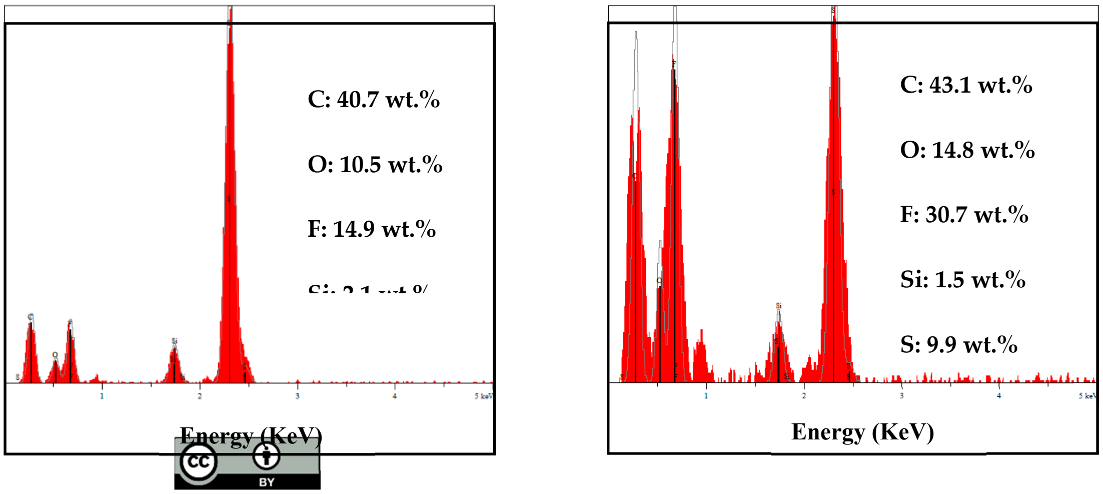

Copyright: © 2021 by the authors. Licensee MDPI, Basel, Switzerland. This article is an open access article distributed under the terms and conditions of the Creative Commons Attribution (CC BY) license (https://creativecommons.org/licenses/by/4.0/).

(a) (b)

Figure S1. EDX analyses of (**a**) SiO_2/(PTFE/PEI)$_5$/PPS and (b) SiO_2/(PTFE/PEI)$_{10}$/PPS composite filter media.

Table S1. BET surface areas and BJH adsorption summary pore volumes of the PPS filter medium and $SiO_2/(PTFE/PEI)_{10}/PPS$ composite filter medium.

Filter media	BET surface area ($m^2\ g^{-1}$)	BJH adsorption summary pore volume ($cm^3\ g^{-1}$)
PPS	4.917	0.004
$SiO_2/(PTFE/PEI)_{10}/PPS$	24.419	0.034

Table S2. Wear resistance of the filter media after measuring.

Filter media	Wear resistance (times)
PPS	60
$SiO_2/(PTFE/PEI)_{10}/PPS$	121

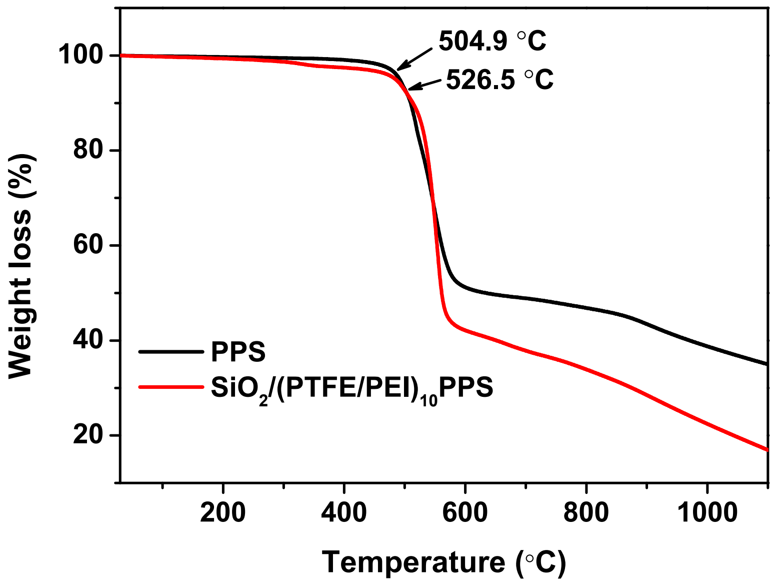

Figure S2. TG curves of the PPS filter medium and $SiO_2/(PTFE/PEI)_{10}/PPS$ composite filter medium after measuring.
